# Two Pathogenic Fungi Isolated From Chalkbrood Samples and Honey Bee Viruses They Carried

**DOI:** 10.3389/fmicb.2022.843842

**Published:** 2022-04-12

**Authors:** Xuefen Cheng, Li Zhang, Ji Luo, Sa Yang, Yanchun Deng, Jianghong Li, Chunsheng Hou

**Affiliations:** ^1^College of Animal Science (College of Bee Science), Fujian Agriculture and Forestry University, Fuzhou, China; ^2^Institute of Bast Fiber Crops, Chinese Academy of Agricultural Sciences, Changsha, China; ^3^Institute of Apicultural Research, Chinese Academy of Agricultural Sciences, Beijing, China; ^4^Guangxi Zhuang Autonomous Region Forestry Research Institute, Nanning, China

**Keywords:** chalkbrood, *Ascosphaera apis*, *Aspergillus tubingensis*, honeybee viruses, transmission mode

## Abstract

*Ascosphaera apis* and some *Aspergillus* species are the main pathogenic fungi of honey bee, and *A. apis* is the pathogen of chalkbrood disease. However, the infection mechanism of them is incompletely known and it is still unclear whether other factors impact their pathogenesis. In this study, *Aspergillus tubingensis* were obtained from the chalkbrood bee samples for the first time. Our results showed that *A. tubingensis* could promote the accumulation of the spores of *A. apis*. Pathogenicity test found that inoculation of the spores of the two fungi alone or their combination could induce disease characterization of chalkbrood and stonebrood but the extent was less than those in field. To further identify other pathogens impacted the pathogenesis, we found several honey bee viruses presented in the pathogenic fungi *A. apis* and *A. tubingensis*, which were different from previous reported. Our results indicated that acute bee paralysis virus (ABPV) and chronic bee paralysis virus (CBPV) could replicate in these two fungi and increased in titer with the going of cultivation time. In addition, CBPV could not only transmit vertically to the next generation by spores, but also spread horizontally to different fungi through hyphal anastomosis. These results suggested that the honey bee chalkbrood contained the other pathogenic fungi besides *A. apis*, the interactions between different pathogens of chalkbrood microbial communities may influence the prevalence of chalkbrood. Moreover, the discovery of honey bee viruses and their transmission mode in these two fungi enhanced the potential of exploring fungi virus as valuable factors that cause fungal disease outbreak.

## Introduction

Honey bees are not only one of the main pollinators in natural ecosystems, but also have great significance to global agricultural development, food crop security, and ecological diversity (Burkle et al., 2013; Garibaldi et al., 2016). However, honey bee colony health is affected by various factors, including pathogenic bacteria, fungi, viruses, parasites, and pesticides (Cox-Foster et al., 2007; Bacandritsos et al., 2010; Dainat et al., 2012). Among them, viruses not only affect honey bee behavior and shorten honey bee life, but also spill over to other insects including wild bumblebees, wild bee species, hoverfly, *Exoneura* spp., *Halictidae* spp., and so on (Bailes et al., 2018; Alger et al., 2019; Tapia-Gonzalez et al., 2019; Brettell et al., 2020).

Chalkbrood disease is caused by a fungal pathogenic *Ascosphaera apis* in honey bees (*Apis mellifera* L.; Spiltoir and Olive, 1955) and exclusively affects honey bee brood. The typical symptom is that there are several colors of chalkbrood mummies, such as white, brown, or black, which depends on the presence of ascospores (Aronstein and Murray, 2010). It is hypothesized that the color is determined by the interactions among fungi or uneven distribution of fungi in the environment (Christensen and Gilliam, 1983). *Aspergillus* fungi are ubiquitous in the environment of honey bees and are facultative pathogens that infect honey bees. A variety of *Aspergillus* species can be isolated from stonebrood samples and environment of colony (Shoreit and Bagy, 1995; Zidan et al., 1999). Hyphal tip isolation and single-spore separation are usually adopted to isolate *A. apis* strain (Spiltoir, 1955; Jensen et al., 2013; Li et al., 2014). Therefore, it is speculated that there may be an interaction between *A. apis* and some *Aspergillus* species in the honey bee gut, which might affect the morphology and prevalence of chalkbrood disease.

A previous study has found that honey bee viruses could infect and replicate in the fungal pathogen *A. apis* (Li et al., 2014). Mycoviruses are a class of viruses that can infect and replicate in fungi, which include dsRNA, ssRNA, dsDNA, and ssDNA viruses according to the genome classification (Kotta-Loizou, 2021). The transmission mode is an important characteristic of mycoviruses and includes horizontal transmission and vertical transmission. Mycoviruses without a spore transmission pathway can be transmitted horizontally through hyphal anastomosis, but most of them are confined to vegetative compatible fungi, and/or vertically transmit to fungal offspring by sexual or asexual spores (Buck, 1998; Wang et al., 2013). Therefore, spores play an important role in the transmission of mycoviruses and most asexual spores can carry the virus, while the transmission ability of virus in sexual spores is weak (Polashock et al., 1997; van Heerden et al., 2001).

Here, we speculated whether there were other “cryptic” fungi in chalkbrood, such as *Aspergillus* species, which interacted with *A. apis* to affect the morphology and prevalence of chalkbrood. Moreover, it had not been studied whether there are still other honey bee viruses in honey bee pathogenic fungi. In present study, we obtained and identified two pathogenic fungi, *A. apis* and *A. tubingensis*, in chalkbrood samples for the first time, and then, we found *A. tubingensis* promoted the accumulation of the spores of *A. apis*. Moreover, chronic bee paralysis virus (CBPV), acute bee paralysis virus (ABPV), Israeli Acute Paralysis Virus (IAPV), Kakugo virus (KV), and Deformed wing virus (DWV) were identified in *A. tubingensis*, while CBPV and ABPV in *A. apis*. It was also found that ABPV and CBPV not only replicated in *A. tubingensis* and *A. apis*, but also their titer was increased with the going of cultivation time. These studies provide a theoretical basis for the discovery of cryptic pathogenic fungi, and fungi as virus transmission vector and pathogen control in honey bees.

## Materials and Methods

### Chalkbrood and Fungal Samples

Chalkbrood samples of *Apis mellifera* L. were collected from the apiary severely infected with chalkbrood disease in Xinjiang province (N43°19′16.71, E84°01′3.22), China during April 2020. The chalkbrood mummies were collected from the bottom board or beehive entrance, while the surface of the mummies was covered with white filamentous and black spores. Then samples with typical symptoms from 10 colonies were selected, and all samples were stored in a refrigerator at −80°C until used for testing. *Aspergillus niger* ATCC16404 was purchased from China General Microbiological Culture Collection Center (CGMCC).

### Isolation and Purification of Fungi

Fungi were isolated and purified as previously described with modifications (Burnside, 1981; Jensen et al., 2013).

Method 1 (hyphal tip isolation): Mummies were soaked in 10% NaClO for 10 min and washed twice in sterile water for 2 min. Then, the surface-sterilized chalkbrood mummies were cut into small pieces, which were further transferred to PDA (Coolaber, Beijing, China) medium with 100 μg/ml of ampicillin and incubated for 3–6 days in the dark at 30°C. The fungi were purified using hyphal-tipping technique under microscope for two rounds and cultured on PDA medium without antibiotic.

Method 2 (single-spore separation): Surface sterilization of chalkbrood mummies was conducted as above described. Then honey bee mummies were chopped up with sterile scalpel and put into new tube containing sterile water. After vortex, spore suspension was gradiently diluted to 10^−2^–10^−4^ and then spread on the PDA medium containing 100 μg/ml of ampicillin, which were incubated at 30°C for 2–3 days in darkness. Under the microscope, a single-spore colony was marked and the corresponding fungal plug was taken to new antibiotic-free PDA medium for further cultivation at 30°C for 4–6 days.

### Morphological Observation of the Fungi

The hyphae block of purified fungi with a diameter of 0.5 cm was cut at the edge of the colony and placed to the center of PDA medium covered by cellophane membrane. After cultivating at 30°C for 2–3 days, the marginal hyphae were cut and observed by microscope. Furthermore, the conidiophore and mature ascoma were gently picked with sterile tweezers and placed on a glass slide with two drops of sterile water, which were directly observed using microscope (Ningbo Sunny Instruments, Zhejiang, China).

### Molecular Identification of Fungal Species

Fungi with different morphology were further identified by amplification of conserved genes containing internal transcribed spacer (ITS) and β-tubulin (for *Aspergillus*; Glass and Donaldson, 1995). Briefly, fungi were cultivated on PDA plates covered by sterilized cellophane membranes at 30°C for 6 days. Then 100 mg mycelia were collected for genomic DNA extraction, which was performed using Fungal Genomic DNA Extraction Kit (Solarbio, Beijing, China) according to the manufacturer’s instructions. DNA amplifications were performed in a 50 μl reaction volume, including 0.5 μM (each) primer (Supplementary Table S1), approximately 100 ng of genomic DNA and 25 μl of 2 × Es Taq MasterMix (CW Biotech, Beijing, China). The *A. niger* amplifications were used as positive controls. The PCR amplification was carried out in a thermocycler (Bioer, China) under the following conditions: 2 min at 94°C, followed by 30 cycles at 94°C for 30 s, 55°C or 58°C for 30 s, and 72°C for 30 s, and 72°C, 5 min for extra extension. PCR products were electrophoresed in 1% agarose gel containing 1 mg/ml GoldView (SBS Genetech Corp. Ltd., Beijing, China) and observed under gel imaging analysis system instrument (MiuLab, GIS-130, Beijing, China). The target gene fragments were purified by gel extraction (CW Biotech, Beijing, China) and verified by DNA sequencing (Sangon Biotech, Shanghai, China). The resulting sequences were analyzed by BLAST at https://blast.ncbi.nlm.nih.gov/Blast.cgi.

### Growth Rate Measurement of Fungi

When the mycelium of purified fungi grew to about 2–3 cm at 30°C, the hyphal plugs was obtained and inoculated as the above described. The inoculated petri dishes were sealed and cultivated at 30°C, and a straight line was drawn across the center of the bottom of the petri dishes to measure and record the colony diameter every 24 h with three replicates in each group.

### Re-inoculation of Spores to Honey Bee Larvae

Spore cysts were produced between two opposing mating types of *A. apis* using mating tests, which were performed as previously described (Jensen et al., 2013). Then the suspension of ascospores was obtained according to the method of Tejerina and Benitez-Ahrendts (2021) with several modifications. Briefly, spore cysts were scraped from the surface of the colony using sterilized inoculating loop and collected in tubes containing sterile water and glass beads. The tubes were vortexed for 30 s (Vortex-Genie 2, Scientific Industries, United States) and centrifuged for 1 min at 15,294 *g*, then discarded the supernatant, and re-suspended the spore precipitate and repeated the steps three times. For obtaining conidia solutions, the procedures were followed according to previously described (Wu et al., 2012). Briefly, when conidia presented in the colonies, they were scraped from the surface of the colony with a sterilized inoculating loop. The conidia mixtures were pooled and filtered through four-layer cheesecloth to remove mycelial fragments. Spores were counted using hemocytometer and the suspensions of spores were adjusted to a concentration of 5 × 10^6^ spores/ml. The spore mixture of *A. apis* and *A. tubingensis* was obtained by mixing spore suspension of *A. apis* and *A. tubingensis* in a ratio of 1:1. New spore solutions were made prior to each experiment. The honey bee larvae (*Apis mellifera* L.) with 1 day old were collected from three random colonies located in the institute of apicultural research of Beijing, China during September and they were fed in sterile 48-well plate as proposed by Schmehl et al. (2016). Spore suspensions (10 μl) were added to the plate well containing 5 days instar larvae and sterile water was used as the control. Each replicate contained four larvae and three replicates in each group. The larvae were cultured at 35°C and 90% humidity, and the larval morphology was observed and recorded every day until 7 days after inoculation.

### Detection of Honey Bee Viruses in Fungi

In order to detect several normal honey bee viruses in fungi, RNA extraction and reverse transcription PCR were conducted. For total RNA extraction, mycelial plugs were inoculated on PDA plates covered by sterilized cellophane membranes at 30°C for 6 days in the darkness. Around 100–200 mg mixture of mycelia and spores were collected and ground into a fine powder in liquid nitrogen. RNA extraction was conducted using ZN Fungal/Bacterial RNA Prep (TIANMO Biotech, Beijing, China) according to the manufacturer’s instructions. The resulting RNA was stored at −80°C before needed. Synthesis of cDNA was performed with 1 μg of total RNA using HiFiscript cDNA Synthesis Kit (CW Biotech, Beijing, China) in the presence of primer mixture (consist of Oligo dT and random primer) according to the manufacturer’s instructions. Then RT-PCR reaction mixture contained 1 μl of forward and reverse primers (Supplementary Table S2), respectively, 10 μl of 2 × Es Taq Master Mix (CW Biotech, Beijing, China), 1 μl of cDNA, and 7 μl of ddH_2_O. PCR was performed with cycling conditions as follows: 94°C for 2 min, followed by 30 cycles at 94°C for 30 s, 55°C for 30 s, and 72°C for 30 s, and 72°C, 5 min for extra extension. Detection, sequencing, and analysis of the expected DNA products were performed as the above description. The sequence data from this study were submitted to and obtained accession numbers from GenBank.

### cDNA Synthesis of Positive and Negative-Strand RNAs

Total RNA was extracted from *A. tubingensis* and *A. apis* using ZN Fungal/Bacterial RNA Prep (TIANMO Biotech, Beijing, China), and cDNA was synthesized using tagged specific primer (Supplementary Table S3) with super RT cDNA Synthesis Kit (CW Biotech, Beijing, China). The cDNA synthesis mixture was incubated at 42°C for 15 min, followed by 85°C for 5 min to terminate reaction. Then RNase H (Solarbio, Beijing, China) was added into the mixture, which was incubated at 37°C for 30 min to degrade RNA.

### Strand-Specific Quantitative Real-Time PCR

In order to quantify the positive and negative-strand RNAs, we first plotted their standard curves. For DNA fragment amplification, 1 μl of the above dilute cDNAs (1:50) were used as templates. The other PCR reagents included non-tagged primer and tag primer (0.5 μM of each, see Supplementary Table S4), 10 μl of 2 × Es Taq MasterMix and ddH_2_O to 20 μl. Thermal cycling protocol was one cycle of 94°C for 2 min followed by 30 cycles of 94°C for 3 s, 57°C for 30 s, 72°C for 30 s, and 72°C for 5 min for extra extension. DNA fragments were recovered using Zymoclean Gel DNA Recovery Kit™ (CW Biotech, Beijing, China), and the purified fragments were cloned into plasmid pMD-18T using pMDTM-18T Vector Cloning Kit (TaKaRa, Japan). Then the constructed plasmids were transformed into competent cells *Escherichia coli* DH5α (Promega, Madison, WI, United States). The recombinant plasmids were extracted using the PurePlasmid MiniPrep Kit (CW Biotech, Beijing, China) and verified by sequencing (Sangon Biotech, Shanghai, China). All the above reactions were performed according to the manufacturer’s recommended instructions. The resulting plasmids for RT-qPCR were diluted to 10^−2^–10^−8^ and reacted with specific forward or reserve primers and tag primer (Supplementary Table S4). Reactions were subjected to 94°C, 3 min followed by 40 cycles of 95°C for 3 s; 60°C for 30 s in the presence of KAPA SYBR FAST qPCR kit (Sigma-Aldrich, United States). The real-time qPCR was performed on LineGene 9600 Plus real-time PCR instrument and the data was analyzed using software version 9600 Plus Software. The forward and reverse primers were designed in NCBI primer blast online,^1^ based on the sequences of ABPV and CBPV submitted to NCBI Gen bank accession number: ABPV (MZ683936) and CBPV (OL321881) of *A. apis*, ABPV (OL321878) and CBPV (OL321879) of *A. tubingensis* in this study. Primer sequence of tag was non-homologous to the honey bee virus as previously described (Yue and Genersch, 2005).

Quantification of the positive and negative-strand RNA was determined using strand-specific RT-qPCR. The contents of mixture (20 μl) were similar to that of DNA amplification for standard curve. The reactions were performed on LineGene 9600 Plus real-time PCR instrument, and the results were analyzed using 9600 Plus Software and standard curves.

### Vertical and Horizontal Transmission of Viruses

In order to test whether virus can be transmitted to offspring or other related virus-free fungi, we followed the protocol of Wu et al. (2012) with several modifications.

To study vertical transmission of viruses, fresh fungal plug was cultivated on PDA plate at 30°C for 5–7 days. When colonies produced conidia or ascospore, the solution of conidia or ascospore was prepared according to the above method. Then 100 μl of diluted spore suspension was spread on the PDA plate, which was incubated at 30°C for 2–4 days in darkness. The mycelia at the edge of a colony were collected for virus detection.

To study horizontal transmission of viruses, pairing culture technique was used. Viruses were transferred to virus-free strain of *A. niger* through hyphal anastomosis. In brief, two different strains were placed on the two sides of PDA plate, respectively, with 2 cm distance. *Ascosphaera tubingensis* and *A. apis* served as the donors, whereas virus-free strain *A. niger* served as recipient. After 17 days under dark condition at 30°C, three mycelium plugs from *A. niger* were taken and placed on the new PDA plates. The mycelia near the edge middle of *A. niger* colony were labeled JL1, mycelia below the edge of *A. niger* colony were labeled JL2, and the mycelia far from *A. tubingensis* colony were labeled JL3. Detection of the viruses in mycelia using the methods above described.

## Results

### Isolation and Purification of Fungi

**Figure 1:**
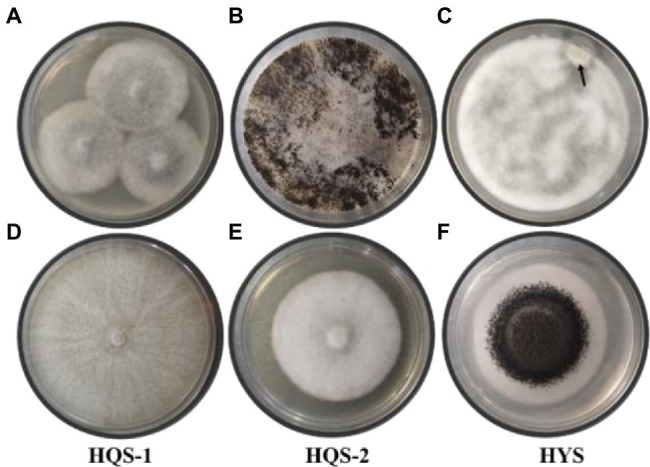
Colonies of preliminarily isolated fungal from chalkbrood samples and colonies of purified fungi. Chalkbrood mummies treated with method 1 and cultured on PDA plate for 6 days **(A)**. Chalkbrood mummies treated with method 1 and cultured on PDA plate for 30 days **(B)**. Chalkbrood mummies treated with method 2 and cultured on PDA plate for 2 days **(C)**. Colonies growing from the hyphal tip isolated from the colony of **(A) (D,E)**. Colony growing from the single spore isolated from the colony of **(B) (F)**.

Hyphal tip isolation and single-spore separation were used to purify the fungi from chalkbrood. The surface-sterilized chalkbrood mummies were cut into small pieces and transferred to PDA for cultivation of 6 days, then colonies with gray white surface were observed (Figure 1A). When the plate was cultured at 30°C for 30 days or longer, black spores appeared and the hyphae were no longer fluffy but appressed on the surface of the medium (Figure 1B), while the spore morphology was different from that of common *A. apis*. Method of single-spore isolation was also adopted. After incubation for 2 days, a smaller special colony was observed (Figure 1C), which produced black spores similar to that in Figure 1B. In order to further isolate and purify fungi, the hyphal tip was picked from the edge of the colony in Figure 1A and cultured on PDA plates. As a result, two fungi with different morphology were isolated and labeled as HQS-1 and HQS-2, respectively (Figures 1D,E). The mycelia of HQS-1 and HQS-2 colony were fluffy and velour, respectively. Black spores were selected from the colonies in Figures 1B,C, diluted, and spread on PDA plate. The resulting fungi marked as HYS (Figure 1F), which had a typical morphology of *Aspergillus* with phialides conidiophores and was speculated to belong to genus *Aspergillus*.

### Microscopic Morphology of Purified Fungi

**Figure 2:**
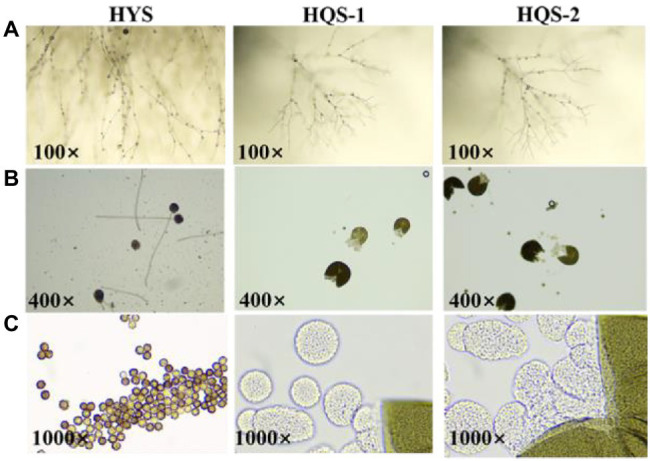
Microscopic morphology of strains HYS, HQS-1, and HQS-2, respectively. Mycelia and spore morphology observed under light microscope (100×; **A**). Conidium and mature ascoma morphology observed under light microscope (400×; **B**). Conidiospore and asci morphology observed under oil lens (1000×; **C**).

Optical microscopy observations showed that most of the hyphae of the three strains were branched (Figure 2A). Besides, the conidia of strain HYS was spherical, and the mature ascoma of strains HQS-1 and HQS-2 were cracked, nearly spherical and hyaline, which contained several asci varied in diameter (Figure 2B). There were a large number of ascospores gathered in asci (Figure 2C). These results indicated that HQS-1and HQS-2 might belong to *Ascomycete*, and HYS might belong to genus *Aspergillus*.

### Molecular Identification of Fungal Species

**Figure 3:**
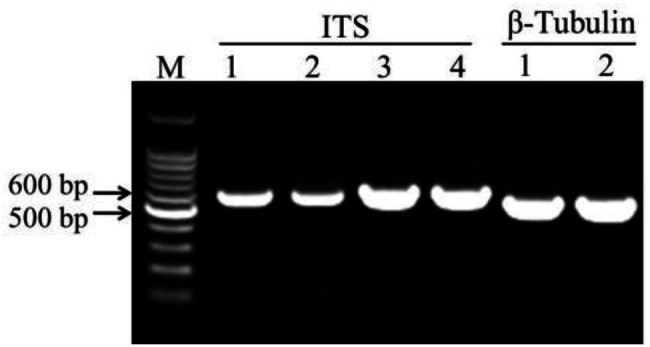
Amplification of conserved gene fragments in filamentous fungi. M: marker. 1. Standard strain *Aspergillus niger*. 2. Strain HYS. 3. Strain HQS-1. 4. Strain HQS-2.

The DNA fragments of ITS were amplified for molecular identification of fungal species (Figure 3). The ITS sequence alignments showed that strain HYS had 99.29% similarity with *A. tubingensis* isolate TF1 and *A. niger* isolate NRF9. In addition, the similarity of strain HQS-1 and HQS-2 to *A. apis* isolate SX and *A. apis* strain CBS534.69 was both up to 100%. In order to further identify strain HYS, β-tubulin was amplified and analyzed. The alignment showed that the strain HYS had 100% similarity to the first 16 *A. tubingensis* species. These results combined with morphological analysis suggested that strain HYS was *A. tubingensis*, strains HQS-1 and HQS-2 were *A. apis*.

### Growth Rate and Pathogenic Characteristics of Fungi

**Figure 4:**
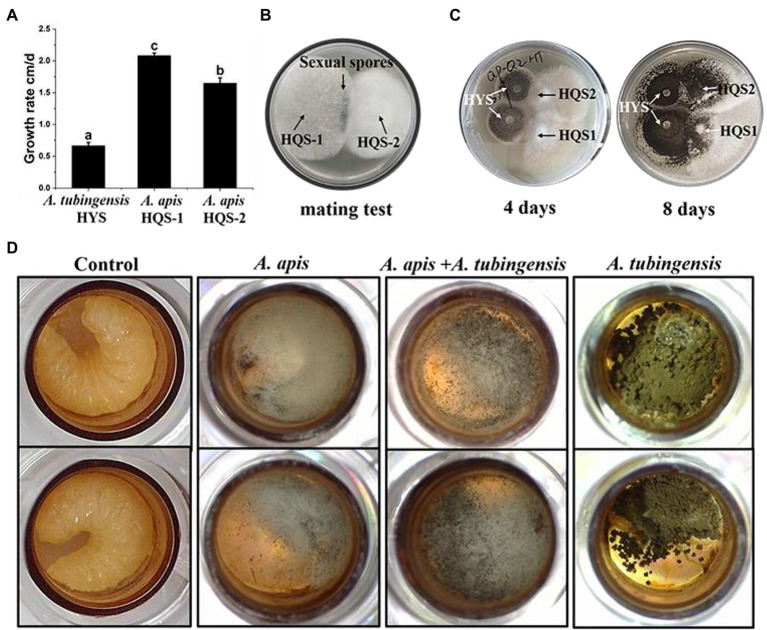
Growth rate and pathogenicity test of *Aspergillus apis* and *Aspergillus tubingensis*. Growth rate of *A. apis* and *A. tubingensis* **(A)**. Mating test between *A. apis* HQS-1 and HQS-2 **(B)**. Co-cultivation of *A. apis* and *A. tubingensis* on PDA plate at different culture time **(C)**. *Aspergillus apis* and *A. tubingensis* inoculated honey bee larvae alone or together **(D)**.

Measurement of the growth rate showed that *A. apis* HQS-1 grew faster than *A. apis* HQS-2, and both of them grew faster than *A. tubingensis* HYS. Furthermore, the growth rates of *A. tubingensis* HYS, *A. apis* HQS-1, and HQS-2 were 0.67, 2.08, and 1.65 cm/days, respectively (Figure 4A). Sexual spores were produced between the opposing mating types of *A. apis* HQS-1 and HQS-2 (Figure 4B). When *A. apis* HQS-1, HQS-2, and *A. tubingensis* HYS were co-cultured on PDA medium, the contact of hyphae could be observed after 4 days. While after 8 days of culture, mycelia of *A. tubingensis* HYS covered the surface of *A. apis* colonies (Figure 4C). Three groups of honey bee larvae were infected with the spores of *A. apis*, *A. tubingensis* alone or combination, the results showed that 33.33–41.67% of honey bee larvae in each group were infected (Supplementary Table S5). Although the mortalities were similar among the three groups, the characteristics of infected honey bee larvae differed significantly. There were many white mycelia and a small number of ascospores on the surface of honey bee larvae infected with *A. apis*. On the contrary, a large number of gray black conidiospores covered on the surface of honey bee larvae infected with *A. tubingensis*. However, after co-infection with spores of *A. apis* and *A. tubingensis*, there were a large number of white hyphae on the surface of honey bee larvae, and the number of spores was much more than that infected with *A. apis* alone, meanwhile, the infected larvae did not show symptom of *A. tubingensis* infection (Figure 4D). These results indicated that both *A. apis* and *A. tubingensis* could infect honey bee larvae and cause corresponding symptoms. While different from the co-culture in PDA medium, *A. apis* that colonized honey bee larvae had more growth advantages than *A. tubingensis*, or the growth of *A. tubingensis* was inhibited by *A. apis* in honey bee larvae gut. It might be further indicated that the presence of *A. tubingensis* in honey bee larvae could promote the spore production of *A. apis* and the prevalence of chalkbrood.

### Detection of Honey Bee Viruses

**Figure 5:**
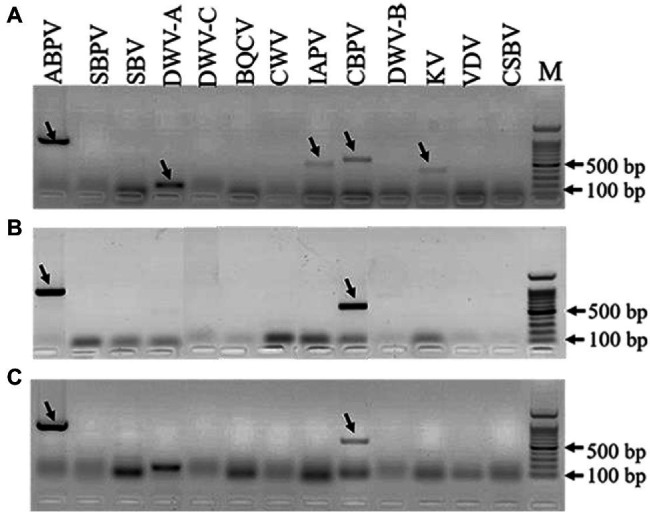
Detection of honey bee viruses in *Aspergillus tubingensis* and *Aspergillus apis*. Viruses detection in *A. tubingensis* HYS **(A)**. Virus detection in *A. apis* HQS-1 and HQS-2, respectively **(B,C)**. M: 100 bp DNA Marker. The arrowheads indicated the destination strips. To avoid potential recombination between Deformed wing virus (DWV) and VDV, we used another pair of primers to detect the presence of VDV.

The results of virus amplification in *A. tubingensis* and *A. apis* were shown in Figure 5. We detected several honey bee viruses in both *A. tubingensis* and *A. apis*. ABPV (accession number: OL321878), CBPV (accession number: OL321879), IAPV (accession number: OL321884), KV (accession number: OL321882), and DWV-A (accession number: OL321883) were detected in *A. tubingensis*. Besides, ABPV (accession number: MZ683936) and CBPV (accession number: OL321881) were detected in both *A. apis* HQS-1 and HQS-2. There was no difference in virus species between *A. apis* HQS-1 and HQS-2.

### Quantification of ABPV and CBPV

**Figure 6:**
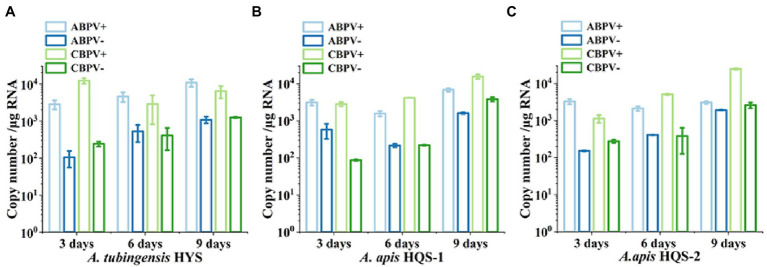
Quantify the positive and negative-strand RNAs of acute bee paralysis virus (ABPV) and chronic bee paralysis virus (CBPV) in *Aspergillus tubingensis* and *Aspergillus apis* at different culture times. Copy number of positive and negative-strand RNAs of ABPV and CBPV in *A. tubingensis* **(A)**. Copy number of positive and negative-strand RNAs of ABPV and CBPV in *A. apis* HQS-1 and HQS-2, respectively **(B,C)**.

As both ABPV and CBPV were detected in *A. apis* and *A. tubingensis* with very obvious bands, the replication and accumulation of which were tested in *A. apis* and *A. tubingensis* using strand-specific RT-qPCR. Hence, the presence of negative-strand RNA and positive-strand RNA for each virus would be determined when fresh fungal plugs were cultivated on PDA for 3, 6, and 9 days. The specificity of positive and negative chain amplification primers of ABPV and CBPV and the standard curves had been verified (Supplementary Figure S1). As it was showed, with the increase of culture time, ABPV titers increased in *A. tubingensis*, while the titers of CBPV were high on the third day then decreased slightly (Figure 6A). Meanwhile, the replication levels of ABPV and CBPV in *A. tubingensis* increased gradually, indicating that ABPV and CBPV could exist and replicate in *A. tubingensis*. For *A. apis*, ABPV titers of HQS-1 and HQS-2 both slightly decreased and then increased, and CBPV titers steadily increased from day 3 to day 9 (Figures 6B,C). Similarly, the replication levels of ABPV and CBPV also increased gradually with the exception that the copy number of ABPV in *A. apis* HQS-1 decreased to the lowest on the 6th day, but increased to the highest on the 9th day (Figures 6B,C). The above results suggested that ABPV and CBPV could exist and replicate in *A. apis*, and there was no significant difference in virus titer and replication level between *A. apis* HQS-1 and HQS-2.

### Analysis of Virus Vertical Transmission

Viruses can be transmitted vertically by spores. In order to study the transmission mode and ability of honey bee viruses in *A. tubingensis* and *A. apis*, we detected the virus distribution in the spores of *A. tubingensis* and *A. apis*. Virus detection in spores showed that in *A. tubingensis*, not all viruses could be transmitted to the next generation through spores. CBPV (19/19) could be detected in all spores, which may be beneficial to the long-term stable existence of the virus, while ABPV (12/19), IAPV (7/19), KV (5/19), and DWV-A (4/19) distributed in spores to varying degrees (Table 1) and the virus distribution details was listed in Supplementary Table S6. As for *A. apis*, CBPV (15/15) could be also detected in all spores, while ABPV (10/15) distributed in partial spores (Table 1) and the virus distribution details was listed in Supplementary Table S7. The above results indicated that not all of the five viruses were distributed in the offspring spores, and the vertical transmission ability was CBPV > ABPV > IAPV > KV > DWV-A.

**Table 1:** Detection of viruses in offspring spores of *Aspergillus tubingensis* and *Aspergillus apis*.

Honey bee viruses	*Aspergillus tubingensis*			*Aspergillus apis*		
	Tested conidia	Conidia containing corresponding viruses	Transmission rates	Tested ascospore	Ascospore containing corresponding viruses	Transmission rates
ABPV	19	12	36.16%	15	10	66.67%
CBPV	19	19	100%	15	15	100%
KV	19	5	26.32%	0	0	0
DWV-A	19	4	21.05%	0	0	0
IAPV	19	7	36.84%	0	0	0

### Analysis of Virus Horizontal Transmission

**Figure 7:**
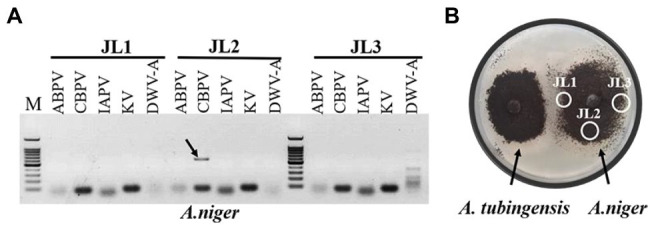
Virus detection in mycelia of *Aspergillus niger*. Virus detection in mycelia of *A. niger* anastomosed with *Aspergillus tubingensis* **(A)**. Hyphal anastomosis between *A. tubingensis* and *A. niger* **(B)**. M: 100 bp DNA Marker.

Viruses also can be transmitted horizontally *via* hyphal anastomosis, so we detected virus distribution in the mycelia of *Aspergillus niger* anastomosed with that of *A. tubingensis* and *A. apis*, respectively. Virus detection in mycelia showed that only CBPV was detected in the mycelia of *A. niger* anastomosed with *A. tubingensis* (Figure 7; Supplementary Table S8). And as expected, no virus was detected in the mycelia of *A. niger* anastomosed with *A. apis* (Supplementary Table S8). Besides, in order to avoid the contamination of *Aspergillus tubingensis*, the strain JL2 was further identified using the primers of β-tubulin (Supplementary Figure S2A). The result sequence was aligned with β-tubulin sequence of standard strain *Aspergillus niger*, and the two sequences were the same, except for the unstable bases generated by sequencing at the two ends (Supplementary Figure S2B), which indicated that strain JL2 was *Aspergillus niger*. The above results suggested that only CBPV could break through the barrier of vegetative incompatibility and be transmitted from *A. tubingensis* to *A. niger* through hyphal anastomosis.

## Discussion

Chalkbrood caused by *A. apis* has been extensively studied. In this study, two kinds of pathogenic fungi were isolated from chalkbrood samples. By directly cultivating slices of chalkbrood mummy or single-spore separation, *A. tubingensis* was isolated. However, when using the former method, *A. tubingensis* needed to be cultured for 30 days or even longer to grow, but *A. tubingensis* could grow in 2 days with single-spore isolation. It was suggested that when *A. apis* and *A. tubingensis* coexisted in honey bee gut environment, the growth of *A. apis* was dominant, while the growth of *A. tubingensis* was restrained, which was consistent with the results of pathogenicity test in this study (Figure 4D). However, after 30 days of cultivation, the growth of *A. apis* might be restricted due to nutrient consumption, thus the inhibitory effect of *A. apis* on *A. tubingensis* weakened, and as a result, *A. tubingensis* grew. When the single-spore isolation method was adopted, the spore suspension was diluted, away from the honey bee intestinal environment and directly cultured on the PDA plate. In this case, the *A. apis* basically had no effect on the growth of *A. tubingensis*. This result was consistent with that of co-culture of the two strains on PDA plate (Figure 4C), the growth of *A. apis* and *A. tubingensis* were not affected by each other. The above results demonstrated that *A. apis* could be isolated by using the traditional method of hyphal tip isolation and obtained by culturing for about a week (Jensen et al., 2013), while it was likely to ignore other cryptic fungi with limited growth. Therefore, it was recommended to culture for a longer time or using single-spore method to isolate other possible fungi from chalkbrood mummies. In fact, *A. tubingensis* was also isolated from chalkbrood samples collected in Fujian province using methods of hyphal tip isolation and single-spore isolation, which indicated that the isolation of *A. tubingensis* from chalkbrood sample was not individual or accidental.

*Aspergillus apis* is a specialized obligate pathogen of honey bee brood. While *Aspergillus tubingensis* is a black *Aspergillus* belonging to the *Aspergillus* section *Nigri*, which includes species that morphologically resemble *Aspergillus niger*. *Aspergillus tubingensis* is widely distributed as human and plant opportunistic pathogen. Infections of *A. tubingensis* in humans have been reported, such as keratomycosis (Kredics et al., 2009), skin infection (Frias-De-Leon et al., 2018), otomycosis (Sabz et al., 2019), and invasive pulmonary aspergillosis (Harada et al., 2020). Besides, it is reported that *A. tubingensis* can also cause fruit rots of grapes (Garcia-Cela et al., 2014), strawberries (Palmer et al., 2019), pomegranate (Guo et al., 2021a), and leaf spot diseases on Jatropha (Guo et al., 2017), Helleborus (Liaquat et al., 2019), and cotton (Khizar et al., 2020). Although a previous study has reported that several *Aspergillus* species, such as *Aspergillus flavus*, *Aspergillus nomius*, and *Aspergillus phoenicis*, were pathogenic to honey bee larvae (Foley et al., 2014), this study was the first report that *A. tubingensis* could infect insect and was the pathogen of honey bee larvae.

On the other hand, *A. flavus*, which is another species of *Aspergillus*, was reported as opportunistic, facultative, but deadly pathogens of honey bees (Vojvodic et al., 2011). *Aspergillus tubingensis* might share similar pathogenic characteristics with *A. flavus* as they are both belonging to the *Aspergillus* section *Nigri*. However, when co-infection of *A. apis* and *A. tubingensis* to honey bee larvae, only symptoms of chalkbrood with much more spores was observed and the growth of *A. tubingensis* was inhibited in the presence of *A. apis*, which might result in *A. tubingensis* was confined to the inside of larvae gut and cannot break through to the surface of larvae as well as promoted the epidemic of chalkbrood. Hence *A. tubingensis* served as cryptic pathogen or promoting factor in this situation, which might explain why *A. tubingensis* could be isolated from chalkbrood samples without symptom of stonebrood.

In practice, there are few reports of stonebrood, but many reports of chalkbrood. Both *A. apis* and *Aspergillus* are transmitted by spores, resulting in the diseases of honey bee larvae. When honey bee larvae ingested the two kinds of spores simultaneously, it was likely that the growth of *Aspergillus* was limited, and the growth of *A. apis* had more advantages, leading to more outbreaks of chalkbrood. Hence, *Aspergillus* spores might be cryptic and exist for a long time in honey bee gut, which was not easy to be found. Once the conditions were suitable, *Aspergillus* was likely to break out, which needed to attract the attention of researchers and beekeepers. Besides, in the intestinal condition, the interaction between *A. tubingensis* and *A. apis* was also worth of further study. In addition, it had been reported that *A. tubingens* had the ability to produce ochratoxin A (OTA; Medina et al., 2005) that might be toxic to honey bees, weaken host immunity and provide favorable conditions for the growth of *A. apis*. Moreover, ochratoxin A produced by *A. tubingensis* might contaminate bee products, which should be paid more attention.

Many studies have reported that mycoviruses are widespread in fungi and yeasts (Herrero et al., 2009; Liu et al., 2014; Zhang et al., 2020; Guo et al., 2021b). Similar to the previous study (Li et al., 2014), we were not only detected IAPV but also CBPV and ABPV. The differences of honey bee virus species might be correlated with the number and region of investigated honey bee colonies, and it was also indicated that more kinds of viruses distributed in honey bee pathogenic fungi. Thus, we advocated a new possibility that fungi might be considered as a honey bee virus vector that tentatively or stably accommodates them.

The mode of transmission is one of the main characteristics of the virus, and the transmission ability determines the spread range and capacity of virus. In this study, vertical transmission test of honey bee viruses showed that only CBPV could spread to the next generation through asexual and sexual spores with the probability of 100%. Horizontal transmission test showed that CBPV was the only honey bee virus that could spread across species from *A. tubingensis* to *A. niger*. A previous study has shown that the horizontal transmission of mycovirus was related to compatibility of vegetative hypha (Zhang and Nuss, 2016). However, some viruses can break through the restriction of vegetative incompatibility and horizontal transmission can occur during some types of incompatible reaction (Deng et al., 2002; Boland, 2004). Therefore, CBPV broken through the restriction of vegetative incompatibility and was transmitted horizontally crossing different species in view of *A. tubingensis* and *A. niger* are closely related species. Several studies have reported that viruses were transmitted across different taxonomic kingdoms. Liu et al. (2016) found that a fungal virus of *Sclerotinia sclerotiorum* hypovirulence associated DNA virus 1 (SsHADV-1) could infect an insect and use it as a transmission vector. Furthermore, it was also discovered that Cucumber mosaic virus (CMV) was transmitted between plants and fungi, and in the process of plant virus transmission, fungi were acted as vectors (Andika et al., 2017). *Aspergillus apis* could infect honey bee larvae, bumblebee larvae and contaminate pollen. Moreover, *A. tubingensis* is human and plant opportunistic pathogen, and *A. niger* is one of the most important microorganisms used in biotechnology, which is also reported as pathogen of human and plants (Schuster et al., 2002; Huang et al., 2020; Mannix et al., 2020; Ghuffar et al., 2021). In this research, CBPV existed and replicated in *A. apis* and *A. tubingensis* and was also transmitted from *A. tubingensis* to *A. niger*. Besides, when analysis the evolution of CBPV, it was found that CBPV was more closely related to Nodavirus, Tombusvirus, and Sclerophthora macrospora virus A (SmVA), and the above viruses belong to insect or fish viruses, plant viruses and fungal viruses, respectively (Ribiere et al., 2010). Hence, whether CBPV uses fungi as vectors for cross-kingdom transmission is worthy of in-depth study.

In conclusion, *A. apis* combined with *A. tubingensis* could induce the outbreak of chalkbrood through pollination and human activities and provide a new way for cross-regional and cross-species transmission of the virus. The host range of honey bee viruses, especially CBPV, and their effects on host are also worth for further study.

## Data Availability Statement

Supplemental data for this article can be accessed at: https://doi.org/10.6084/m9.figshare.19367150.v1.

## Author Contributions

CH and XC conceived this manuscript. XC, JLu, SY, and YD performed the experiments. XC and LZ analyzed the data and wrote the manuscript. CH, LZ, and JLi revised the manuscript. All authors contributed to the article and approved the submitted version.

## Funding

We thank for the support by the Agricultural Science and Technology Innovation Program (CAAS-ASTIP-2019-IAR) and Key Technology Research and Development Program of Guangxi province (AB16380094).

## Conflict of Interest

The authors declare that the research was conducted in the absence of any commercial or financial relationships that could be construed as a potential conflict of interest.

## Publisher’s Note

All claims expressed in this article are solely those of the authors and do not necessarily represent those of their affiliated organizations, or those of the publisher, the editors and the reviewers. Any product that may be evaluated in this article, or claim that may be made by its manufacturer, is not guaranteed or endorsed by the publisher.

## Footnotes

^1^ https://blast.ncbi.nlm.nih.gov/Blast.cgi
